# Cell-size space effects on phase separation of binary polymer blends

**DOI:** 10.1007/s12551-022-01001-0

**Published:** 2022-10-25

**Authors:** Miho Yanagisawa

**Affiliations:** 1 Graduate School of Arts and Sciences, Komaba Institute for Science, The University of Tokyo, Komaba 3-8-1, Meguro, Tokyo, 153-8902 Japan; 2 Department of Physics, Graduate School of Science, The University of Tokyo, Hongo 7-3-1, Bunkyo, Tokyo, 113-0033 Japan; 3 Center for Complex Systems Biology, Universal Biology Institute, The University of Tokyo, Komaba 3-8-1, Meguro, Tokyo, 153-8902 Japan

**Keywords:** Phase transition, Membrane, Wetting, Confinement, LLPS

## Abstract

Within living cells, a diverse array of biomolecules is present at high concentrations. To better understand how molecular behavior differs under such conditions (collectively described as macromolecular crowding), the crowding environment has been reproduced inside artificial cells. We have previously shown that the combination of macromolecular crowding and microscale geometries imposed by the artificial cells can alter the molecular behaviors induced by macromolecular crowding in bulk solutions. We have named the effect that makes such a difference the cell-size space effect (CSE). Here, we review the underlying biophysics of CSE for phase separation of binary polymer blends. We discuss how the cell-size space can initiate phase separation, unlike nano-sized spaces, which are known to hinder nucleation and phase separation. Additionally, we discuss how the dimensions of the artificial cell and its membrane characteristics can significantly impact phase separation dynamics and equilibrium composition. Although these findings are, of themselves, very interesting, their real significance may lie in helping to clarify the functions of the cell membrane and space size in the regulation of intracellular phase separation.

## Introduction

Although life is difficult to define, a living cell has at least three characteristics: replication, metabolism, and compartmentalization (Mann 2012). Attempts have been made to produce cell-like systems to elucidate their physicochemical principles. Of the three major characteristics just highlighted, compartmentalization is the most accessible feature to explore in the preparation and observation of cell-like systems. For example, tiny droplets compartmentalized by lipid membranes have been used as the simplest artificial cells (Hamada and Yoshikawa 2012) (Yanagisawa et al. 2014a, b, c) (Ai et al. 2020) (Lim HW, Wortis et al. 2008) (Dimova and Marques 2019).

However, living cells are not only composed of membranes. There are a diverse range of biomolecules enclosed within the membrane at total biomolecular concentrations as high as 50 to 400 mg/mL (Minton 2001). This highly concentrated, highly volume-occupied biomolecular environment (termed macromolecular crowding) contributes to cellular metabolism and replication (Adamski et al. 2020) (Otto 2022) and is known to alter the behavior of biomolecules seen under dilute conditions (Rivas and Minton 2016) (McGuffee and Elcock 2010). One of the leading causes of these alterations is excluded volume, which is the volume within a solution space that is physically inaccessible to other molecules. When concentrated biomolecular solutions are placed in smaller containers (having a larger surface-area-to-volume ratio (*S/V*)), the effective concentration becomes higher because of the increased fraction of excluded volume along the walls of the container (Marenduzzo et al. 2006). Therefore, wall spacing effects should be considered when analyzing macromolecular crowding. However, it is challenging for living cells because their spatial properties, such as density and shape, change spatiotemporally (Boersma, Zuhorn et al. 2015) (Minton 1992) (Minton 1995).

**Fig. 1:**
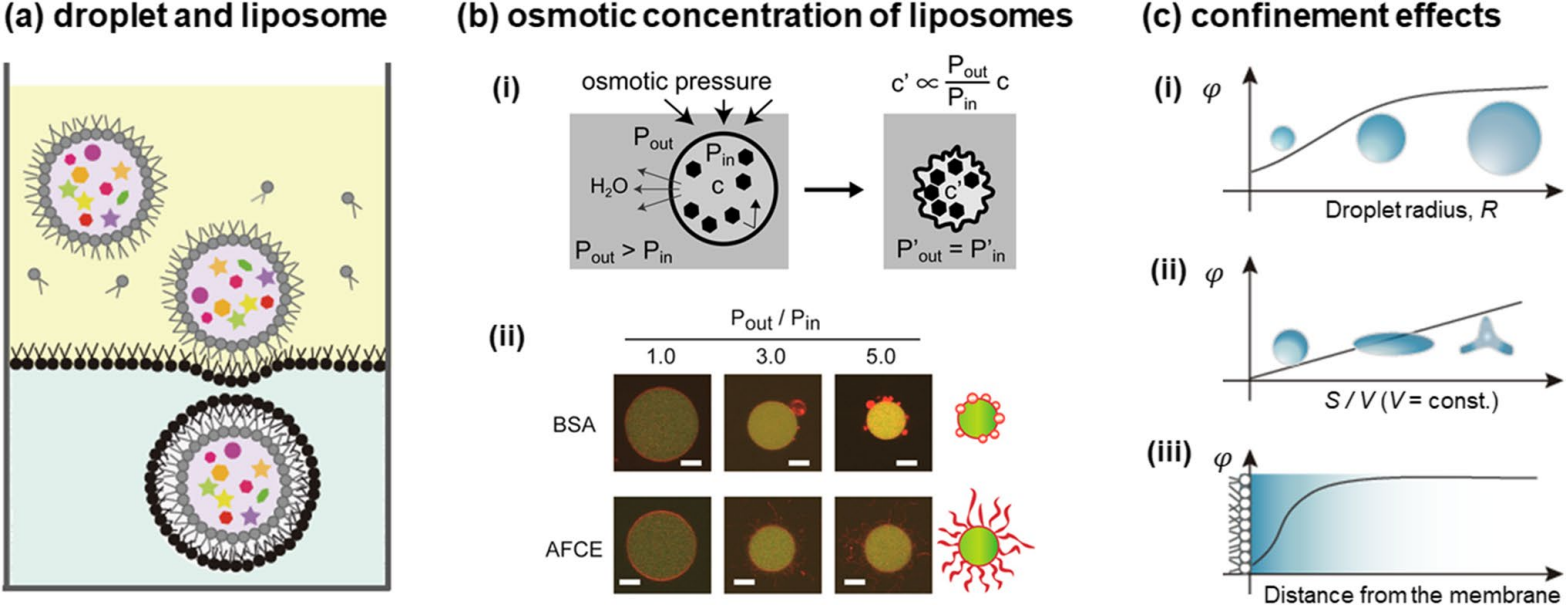
Artificial cells for studying cell-sized confinement effects under macromolecular crowding. (**a**) W/O droplets covered with a lipid layer in the oil phase (upper) and lipid bilayer liposomes in the aqueous phase (lower). (**b**) The hypotonic condensation of biomolecules inside liposomes. (i) As the osmotic pressure ratio between the outer and inner solutions *P*_out_/*P*_in_ grows, the initial concentration of molecules in liposomes *c* rises, reaching a final value, *c*′′, in an isotonic state, *P*_out_/*P*_in_ > 1. (ii) Two liposome deformation patterns that contain BSA or multiple components (an additive-free cell extract, AFCE). Fluorescence images show the deformation of liposomes (red), encapsulating biomolecules, and GFP (green). The schematic images on the right edge show the typical deformation patterns observed for BSA- and AFCE-containing liposomes, i.e., budding and tabulation of membranes. Scaled bars indicate 5 μm. (**c**) Example of graphs to derive main factors in CSEs. A physical property *φ* is plotted against (i) the radius *R* of spherical droplets, (ii) the droplet membrane’s surface-area-to-volume ratio *S/V* under the fixed *V* condition, and (iii) distance *l* from the lipid membrane at the droplet surface. The functional form should be dependent on specific systems and physical properties. Examples of *φ* include the diffusion coefficient *D* (i, iii) and the translation rate *V* (ii). Panel (**b**) is reprinted with permission from Fujiwara and Yanagisawa (2014). Copyright 2014, The American Chemical Society. Panel (**c**) is drawn with reference to Fig. 1 obtained from Yanagisawa, Watanabe et al. (2022), Copyright 2022, The American Chemical Society

Artificial cells have been used to reproduce the macromolecular crowding environment in cells (Fujiwara and Yanagisawa 2014) (Fujiwara, Yanagisawa et al. 2014) (Yanagisawa et al. 2014a, b, c). Examples include liposomes and water-in-oil (W/O) droplets covered with a lipid membrane that encapsulate a concentrated biomolecular solution (Fig. 1). Encapsulated biomolecules inside liposomes have been reported to promote the formation of different molecular structures from those existing in corresponding bulk (Yanagisawa et al. 2014a, b, c) (Miyazaki et al. 2015) (Litschel et al. 2021) (Bashirzadeh et al. 2021). Such observations suggest that spacing effects play a significant role on the structural formation of biomolecules. To quantitatively investigate such spacing effects, biomolecular concentration and spatial parameters, such as cell size, cell shape, and membrane properties, should be controlled independently. In the case of liposomes, we can concentrate the confined biomolecules under a hypotonic condition where the semi-permeable membrane entraps the biomolecules inside (Fig. 1(b), (i)). However, this condensation is accompanied by a variety of membrane deformations depending on the type of molecule encapsulated (Fig. 1), (ii)) (Fujiwara and Yanagisawa 2014) (Liu et al. 2019) (Tanaka, Takiguchi et al. 2018). For example, liposomes encapsulating a solution of bovine serum albumin (BSA) show budding, but the liposomes encapsulating a multicomponent solution of an additive-free cell extract (AFCE) show tabulation (Fig. 1), (ii)). This result means that the size-relating parameters like radius *R* and shape-relating parameters like *S/V* cannot be independently manipulated to derive the space effect (Fig. 1), (i, ii)). In addition, membrane fluctuations occurring within soft liposomes make it difficult to analyze properties with respect to the distance from the membrane (Fig. 1), (iii)).

On the other hand, W/O droplets covered with a lipid layer are relatively easy to independently control in respect to their size, shape, membrane properties, and biomolecule concentration (Hamada and Yoshikawa 2012). This is because the large interfacial tension at the W/O interface stabilizes the system and the outer oil phase prevents molecular transport through the membrane. From our studies using droplets, we found that the small space comparable to a cell significantly alters the various behaviors of encapsulated molecules and we termed this effect the cell-size space effect (CSE) (Yanagisawa, Watanabe et al. 2022). Here, we review both our and others’ published observations of the CSE on phase separation. We start this endeavor by briefly reviewing the basic principles of phase separation in bulk systems (Dill, Bromberg et al. 2010) (Hansen and McDonald 2013). Then, space size–dependent phase separation due to the CSE is presented. Finally, we explain the dominant factors of CSE on the phase separation around the direct and indirect interactions between the encapsulated molecules and the membrane.

## Phase separation in bulk

From a thermodynamics point of view, the basic mechanism of phase separation can be explained by the change in Gibbs free energy due to mixing (Dill, Bromberg et al. 2010) (Hansen and McDonald 2013). Here, we consider the situation where a homogenous solution of a polymer A separates into two liquid phases as the temperature *T* decreases. The change in Gibbs free energy due to mixing *∆G*_mix_ is as follows:

1 $${\Delta G}_{mix}={\Delta H}_{mix}-{T\Delta S}_{mix}$$

**Fig. 2:**
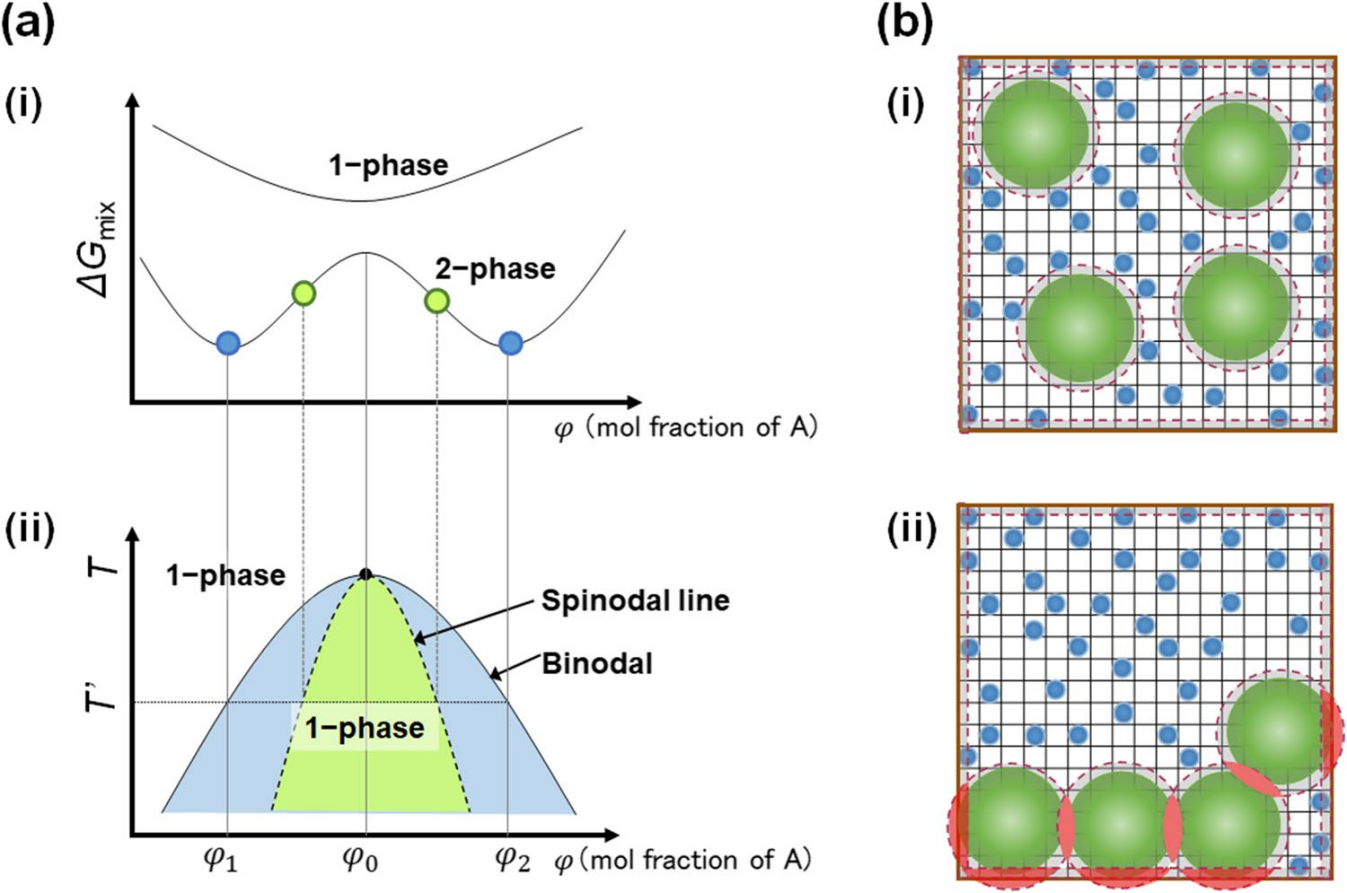
The basic mechanism of phase separation. (**a**) (i) The Gibbs energy change via mixing is plotted against the mole fraction of the polymer at a different temperature. (ii) Phase diagram obtained from the minima of the Gibbs energy change (blue, binodal line) and the inflection point (green, spinodal line). (**b**) A depletion interaction when a small number of large particles (green) coexist with a large number of small particles (blue). (i) The excluded volume at the surface of large particles and the space wall is indicated with a broken line (red). (ii) When large particles aggregate, the excluded regions overlap (red), and the total excluded volume decreases

The first term *∆H*_mix_ is the enthalpy change due to mixing. When the volume change is negligible, *∆H*_mix_ equals the internal energy change that originated from the interaction between different molecules. The second term *TΔS*_mix_ is the corresponding difference in free energy due to entropy change associated with mixing. In Fig. 2) (i), the *∆G*_mix_ is plotted against the mole fraction of polymer A, *φ*, at two different *T*. In the homogeneous single-phase (1-phase) system observed at a higher *T*, *ΔG*_mix_ has a minimum at mole fraction, *φ*_0_. On the other hand, in the coexisting two-phase (2-phase) system observed at lower *T*, *ΔG*_mix_ shows two minima at *φ* = *φ*_1_ and *φ*_2_ (*φ*_1_ > *φ*_2_). By changing *T*, we can derive the phase diagram, which shows the equilibrium composition (Fig. 2), (ii)). The binodal line derived by the minimum points (blue) divides the 1-phase and 2-phase regions, whereas the spinodal line derived by the inflection points (green) classifies the dynamics into two: nucleation-growth type (blue) and spinodal decomposition type (green) (Dill, Bromberg et al. 2010) (Hansen and McDonald 2013).

The combinatorial entropy of mixed ideal polymer solutions was described by Flory and Huggins as a volume fraction, taking into account the size difference between solvent and polymer (Flory 1942) (Teraoka 2002). Increasing the degree of polymerization of the polymer effectively decreases the entropy cost of confining the polymer to the concentrated phase and decreases the concentration at which phase separation occurs (Wang et al. 2019) (Martin and Mittag 2018). On the other hand, entropy gain also results in phase separation in a system composed of a small number of large molecules and a high number of small molecules, as illustrated in Fig. 2) (i). The key to rationalizing this diverse behavior lies in understanding the concept of excluded volume (gray area), a region of space where the molecular center cannot enter and which is defined by its own radius (for symmetrical molecules) (Dill, Bromberg et al. 2010) (Hansen and McDonald 2013). When large molecules assemble in a single component gas, both the component entropy of the large molecule and the system entropy are decreased. In contrast, when these large molecules exist in solution, the overall entropy change of the system generally increases due to the fact that aggregation generally leads to an increase in the free volume, where the small molecules may access and move around. This is because the excluded volume of the large molecule overlaps and decreases (Fig. 2), (ii); red area) (Dill, Bromberg et al. 2010) (Hansen and McDonald 2013). Such depletion type interactions (Asakura and Oosawa 1954) also get stronger as the space becomes narrower and the *S/V* increases because the excluded volume on the space wall increases (Minton 1992) (Minton 1995). In cells, numerous membrane interfaces, including the cellular membrane and membranous organelles, are present. Therefore, while identifying the origin of intracellular phase separation, entropy gain from depletion interactions as well as enthalpy changes from intermolecular contacts must be taken into consideration (Hyman et al. 2014).

## Membrane wetting initiates heterogeneous nucleation

In small systems, such as cell-sized systems, the effects of walls are generally not negligible. Here, we describe the general phenomenon of surface wetting (Kelton and Greer 2010) (Wang et al. 2019). In nucleation- and growth-type phase separation, nuclei first appear in a homogeneous solution as a metastable state which then grow via a process known as homogeneous nucleation. With regard to nucleus growth, any gain of Gibbs free energy associated with the nucleus growth should be overcome by the decrease in interfacial energy as the overall relative surface area (to volume) of the newly formed nucleus decreases. Assuming that the nucleus is spherical with radius *r*, the free energy change for a homogenously nucleating system, *ΔG*_hom_, can be expressed using the free energy change per unit volume *ΔG*_v_ and the interfacial tension *γ* as follows (Kelton and Greer 2010).

2 $${\Delta G}_{\mathrm{hom}}=-\frac{4}{3}{\pi r}^{3}{\Delta G}_{\mathrm{v}}+{4\pi r}^{2}\gamma$$

**Fig. 3:**
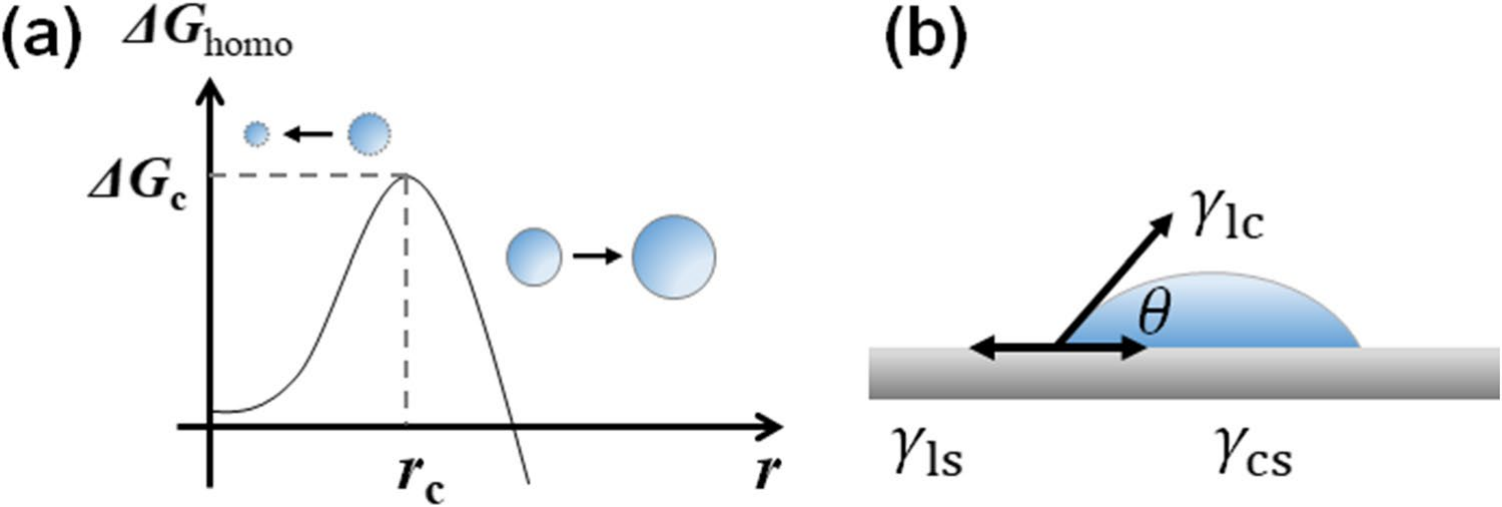
(**a**) Gibbs free energy for homogeneous nucleation *ΔG*_hom_ having a maximum value at the critical nucleus size *r*_c_. (**b**) Schematic of a spherical capped nucleus (s) formed on a substrate (s). The force balance among the three interfacial tensions determines the contact angle *θ.*

Since the first and second terms are proportional to the volume and surface area of the nucleus, Δ*G*_hom_ will have a maximum value at the critical nucleus size *r*_c_ (Fig. 3).

3 $${r}_{c}=\frac{2\gamma }{{\Delta G}_{\mathrm{v}}}.$$

Nuclei larger than *r*_c_ grow in size whereas nuclei smaller than *r*_c_ (called pre-critical nuclei) are thermodynamically unstable, meaning they will melt again (Kelton and Greer 2010). This representation corresponds to the confinement of polymer blends inside small droplets smaller than *r*_*c*_ that inhibits the nucleation and phase separation (Ott and Freedman 2020).

Since the energy barrier at *r*_*c*_ derived from Eqs. (2) and (3) can be significant, most nucleation processes in nature proceed via a so-called heterogeneous nucleation mechanism, in which the wall of the container along with surface impurities promotes the nucleation event (Wang et al. 2019). For example, let us consider the case where a spherical-cap-shaped nucleus (crystal: c) forms at the liquid (liquid: l) substrate (substrate: s) interface (Fig. 3). The value of equilibrium contact angle *θ* is determined by the balance of interfacial tensions between liquid and substrate γ_ls_, liquid and crystal γ_lc_, and crystal and substrate γ_ls_, yielding the so-called Young equation (Kelton and Greer 2010)**.**

4 $$\begin{array}{c}{\gamma }_{\mathrm{ls}}={\gamma }_{\mathrm{cs}}+{\gamma }_{\mathrm{lc}}cos\theta \end{array}$$

If the affinity (wettability) between the nucleus and the substrate is high, the nucleation energy Δ*G*_hom_ lowers by a factor of *f*(*θ*) (Kelton and Greer 2010).

5 $$\begin{array}{c}\Delta {G}_{\mathrm{het}}=\Delta {G}_{\mathrm{hom}}\bullet f\left(\theta \right)\end{array}$$

6 $$\begin{array}{c}f\left(\theta \right)=\left[\frac{1}{2}-\frac{3}{4}\mathrm{cos}\theta +\frac{1}{4}{\mathrm{cos}}^{3}\theta \right]\end{array}$$

When the nuclei exhibit high wettability to the substrate (contact angle ~ 0), *ΔG*_het_ approaches 0, indicating that the higher the wettability, the lower the energy barrier for nucleation. Such wetting contributes to lowering the energy barrier, but does not decrease *r*_*c*_ (Kelton and Greer 2010).

## Phase separation inside the cell-size space

In the above section, we described how membrane wetting can initiate nucleation and growth by lowering the energy barrier. Such acceleration of nucleation and growth has been reported for cell-sized W/O droplets containing binary polymer blends, such as poly(ethylene glycol) (PEG) and gelatin (Yanagisawa et al. 2014a, b, c) (Yamashita, Yanagisawa et al. 2014) and PEG and DNA (Biswas et al. 2012), where the hydrophilic linear polymer PEG behaves as a demixing agent to the biopolymers. In the following, we will explain the dominant factors acting in CSE for depletion-driven and enthalpy-driven phase separation.

**Fig. 4:**
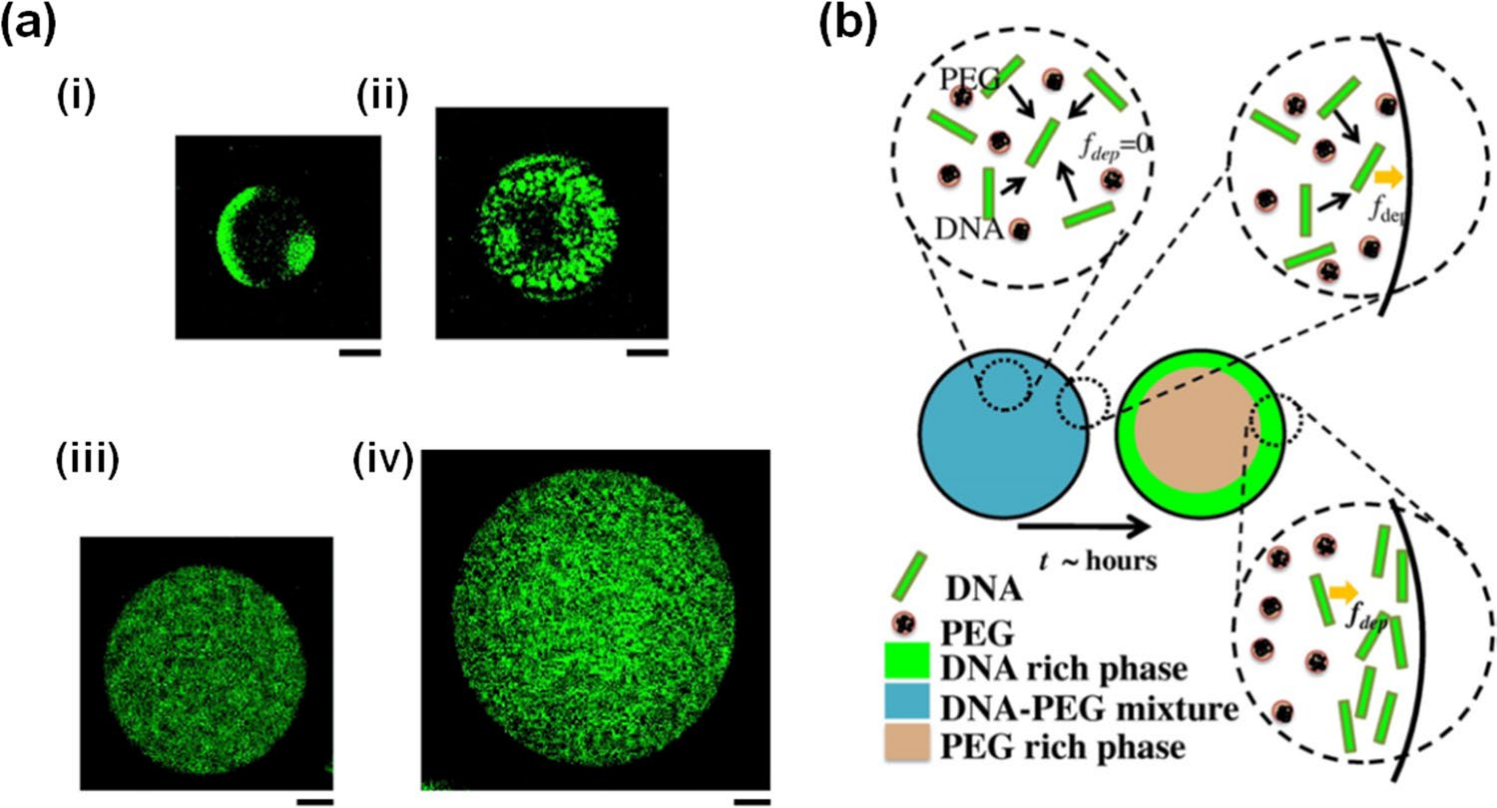
(**a**) Droplet size effect on the phase separation dynamics. Fluorescence microscope images of DNA for binary droplets after 2.5 h of formation (52 mg/mL DNA, 114 mg/mL PEG6k, 71 mM NaCl), where the DNA is stained with the fluorescent dye YOYO-1. The droplet radius *R* is (i) 25 μm, (ii) 39 μm, (iii) 61 μm, and (iv) 85 μm, respectively. Scale bars are 20 μm. (**b**) Schematic of the molecular mechanism for phase separation inside droplets. From the initial homogeneous state (left), DNA molecules tend to attract each other due to the depletion force by the crowded PEG. As a result, the condensed DNA molecules near the droplet surface tend to make a parallel alignment. Panels (**a**) and (**b**) are reprinted with permission from the following reference (Biswas et al. 2012). Copyright 2012 Elsevier B.V

First, we discuss some general observations related to the CSE on depletion-driven phase separation. In this regard, a standard binary solution employed in experimental studies combines a few large DNA and many small PEGs (Biswas et al. 2012) (Gomes et al. 2009). With the surface charge of the DNA screened by the surrounding counter ions, increasing the PEG concentration intensifies the depletion interaction between the DNA molecules. Finally, the solutions begin to separate into two phases (Kojima et al. 2006) (Gomes et al. 2009) (Albertsson 1970). Fig. 4) shows the space size effect on the phase behaviors of 52 mg/mL salmon sperm DNA (300–700 bp), and 114 mg/mL PEG with an average molecular weight of 6000 (PEG6k) at an ionic strength defined by 71 mM NaCl. DNA is a less flexible polymer than the PEG6k as is indicated by the fact that their persistence lengths are approximately 50 nm and 0.37 nm, respectively (Biswas et al. 2012). The variously sized droplets show different states of the phase separation process 2.5 h after the droplet preparation, where the DNA is stained with the fluorescent dye YOYO-1 (seen in green). The smallest droplet with *R* = 25 μm shows macro-phase separation with DNA aggregation near the periphery (Fig. 4), (i)). In droplets with *R* = 39 μm, DNA formed small and multiple nuclei before the macro-phase separation (Fig. 4), (ii)). No visible aggregates of DNA were seen for larger droplets with *R* = 61 µm and 85 µm, but slight density fluctuations of DNA molecules seem to occur (Fig. 4), (iii, iv)). These results demonstrate that such types of phase separation dynamics are accelerated when *R* is less than approximately 40 µm.

To explain the phenomenon of small space–accelerated phase separation, we focus further on the contribution of the depletion interaction (Fig. 4). In relation to the two macrosolutes, significant regions of excluded volume exist near the membrane wall space with this volume region defined by an orthogonal distance that is approximately equal to the particular macrosolutes’ radius of gyration *R*_*g*_ (see Fig. 2). For PEG6k with *R*_*g*_ ~ 3 nm and DNA with persistence length ~ 50 nm, the upper limit radius of droplet size at which phase separation accelerates (~ 40 μm) is quite large. Therefore, the increase in entropy brought about by the walls will be negligible for PEG. Another possible reason is the reduction in enthalpy due to preferential interaction with the membrane covering the droplet surface. Electrical screening of the droplet surface by counterions may also help to reduce the electrostatic repulsion necessary for DNA aggregation (Kato et al. 2009) (Kato et al. 2010). Previous studies considering the elastic properties of droplet surfaces suggest that large molecules are preferentially located at the space’s periphery and center for the cases of hard and soft interfaces, respectively (Shew, Oda et al. 2017) (Dinsmore et al. 1998). In the W/O droplets, such large interfacial tension may effectively act as hard interfaces and contribute to the aggregation of large DNA at the periphery of the droplet.

Such accelerated coarsening upon phase separation inside tiny droplets with *R* < 40 μm has also been observed in PEG and gelatin systems (Yanagisawa et al. 2014a, b, c). However, since there is no significant difference in the characteristic length of PEG and dextran, unlike the above system, the origin may be a hydrodynamic effect. Thus, although the phenomena may result in similar outcomes the underlying molecular driving forces may be different.

**Fig. 5:**
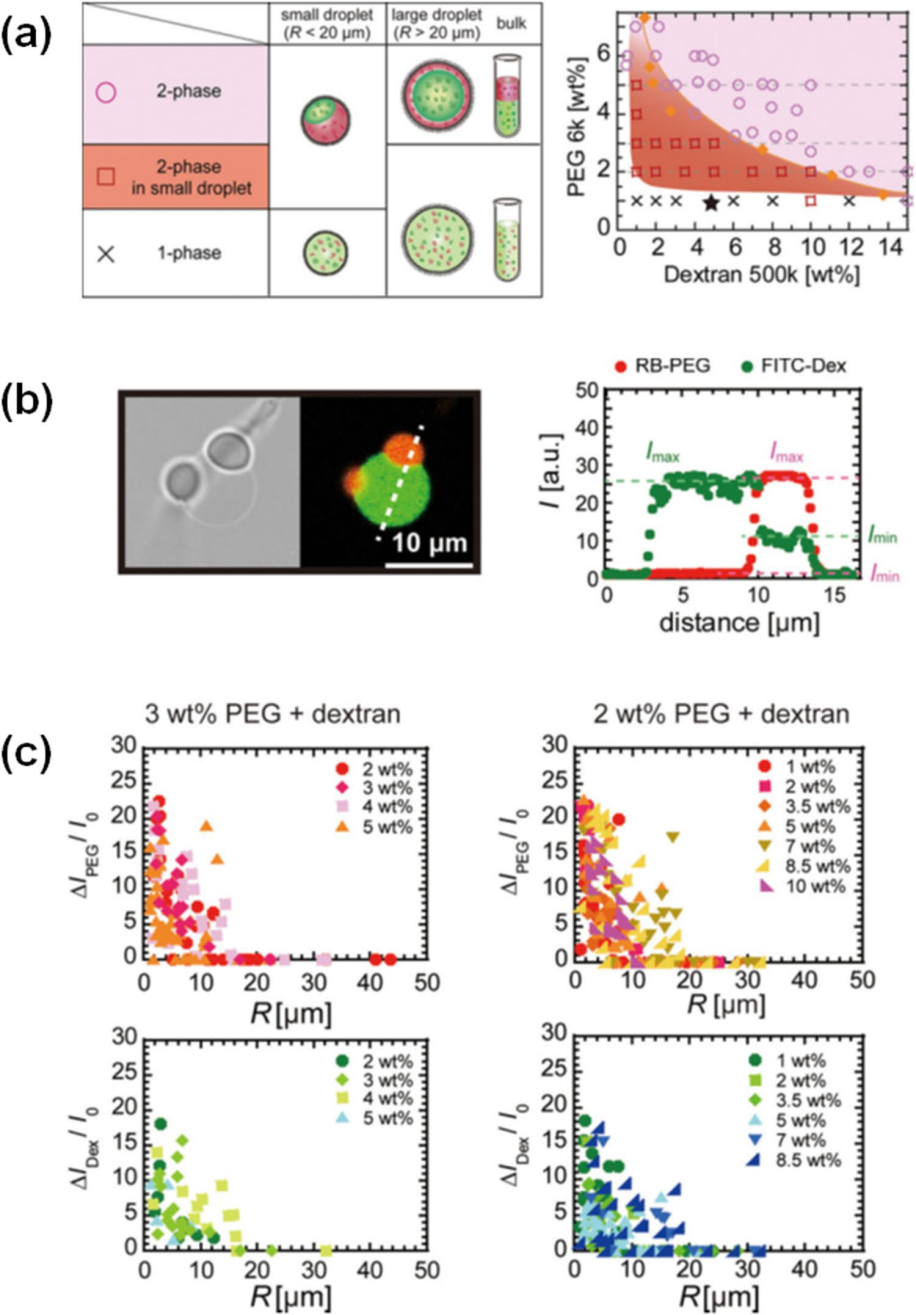
(**a**) Phase diagram of PEG6k and dextran500k blends in droplets with radius *R* ≥ 5 μm. In the 2-phase region (pink, circle), droplets of any size phase separate, which is likely caused by the identical mechanism to that operating in the bulk system. Unlike the case for the 1-phase system (white, cross), strong droplet size–dependent phase separation occurs in the 2-phase region (red, square). (**b**) Transmission (left) and confocal fluorescent image (right) of PC droplet containing 2 wt.% PEG and 7 wt.% dextran with RB-PEG5k (red) and FITC-Dex500k (green). Fluorescence intensity profile shown along the broken line. Fractionation degree evaluated as normalized intensity difference, *ΔI/I*_0_, where *ΔI* = *I*_max_ − *I*_min_ in the droplet of interest and *I*_0_ is the background intensity. (**c**) *R* dependence of *ΔI/I*_0_ of RB-PEG5k (upper) and FITC-Dex500k (bottom) for fixed PEG concentration to be 3 wt.% (left) and 2 wt.% (right) against various dextran concentrations. Panels (**a**), (**b**), and (**c**) are reprinted with permission from Watanabe et al. (2022) Copyright 2022 American Chemical Society.

Next, we discuss our investigations of CSE on the enthalpy-driven phase separation of the binary blends of PEG6k and BSA (Watanabe et al. 2020), or dextran with an average molecular weight of 500,000 (Dextran500k) (Watanabe et al. 2022). To minimize direct membrane interaction with the polymers used in these studies, we employed a zwitterionic lipid, phosphatidylcholine (PC), to cover the droplet surface. In both situations, confined polymer blends only exhibited phase separation in tiny droplets (*R* < 30 μm), even though the equivalent bulk solution exists as a single-phase (1-phase) region. These results show that CSE increases the 2-phase region and moves the critical point to lower concentrations than that observed in bulk systems without CSE (Fig. 5). Furthermore, the extent of phase separation increases with decreasing *R* for PEG6k and BSA or dextran500k systems. To quantify the degree of separation, we analyzed the average intensity of two dyes: rhodamine-B-labeled PEG5k (RB-PEG5k) and fluorescein-isothiocyanate-labeled dextran500k (FITC-Dex500k) which preferentially partitioned into the PEG-rich phase and the dextran-rich phase, respectively. We obtained the maximum and minimum intensities *I*_max_ and *I*_min_ of RB-PEG5k (or FITC-Dex500k) for the PEG-rich and dextran-rich phases (or dextran-rich and PEG-rich phases) (Fig. 5), and plotted the intensity difference normalized by the background, *ΔI*/*I*_0_, where *ΔI* = *I*_max_ − *I*_min_, against the droplet radius, *R*. As shown in Fig. 5), the degree of fractionation into PEG- and dextran-rich phases increases as droplet size decreases for small droplets (*R* ≤ 20 μm).

Here, we discuss the possible mechanisms to explain the observed CSE-induced phase separation. The enhancement of depletion interactions in a small space would be negligible in a blend of two polymers of similar size, stiffness, and molar concentration (as in the present case).

**Fig. 6:**
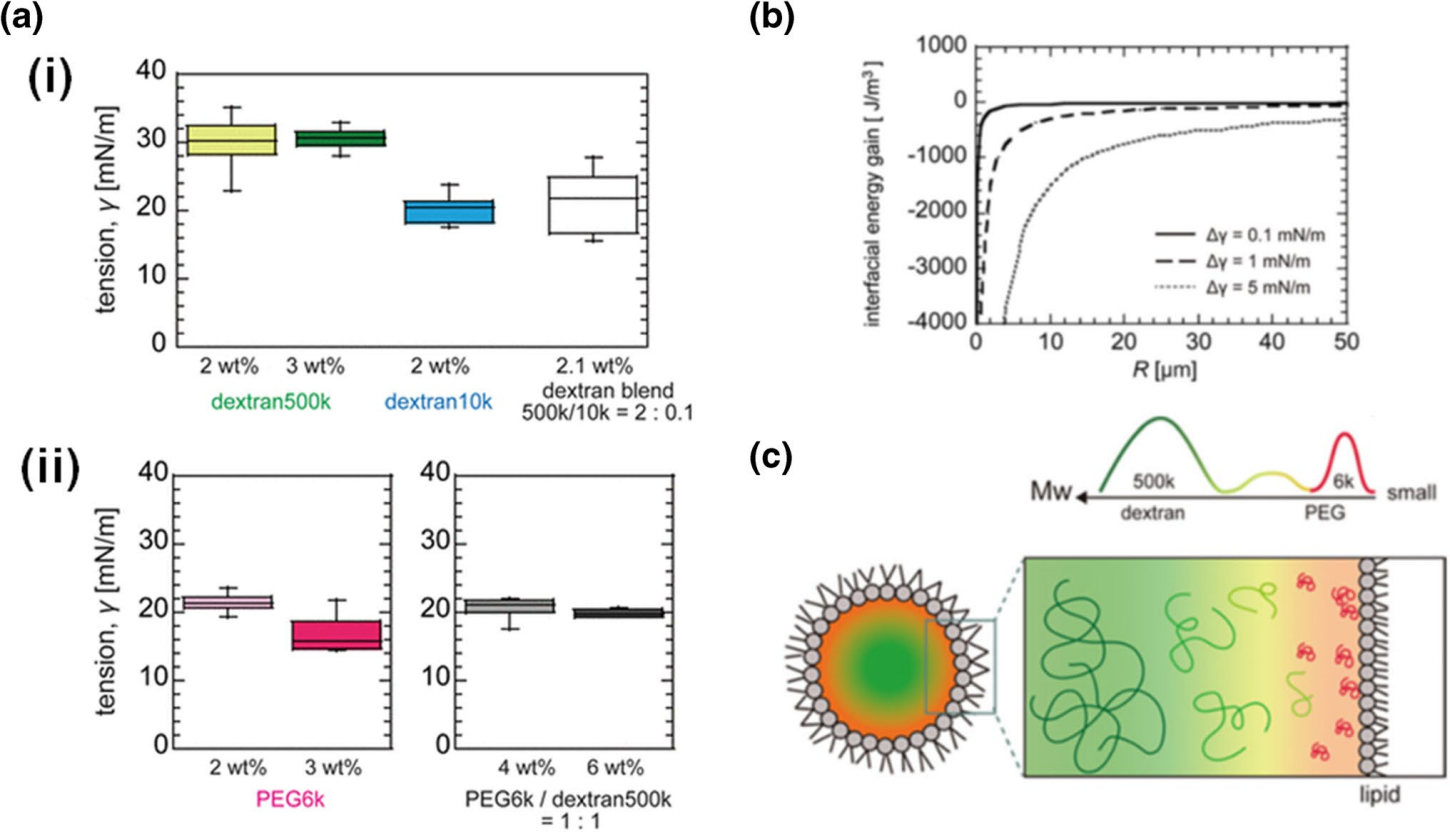
(**a**) Surface tension of PC droplets containing different polymer solutions. (**a**) 2 wt.% dextran500k solution, 3 wt.% dextran500k solution, 2 wt.% dextran10k solution, and a blend of 2 wt.% dextran500k and 0.1 wt.% dextran10k. (**b**) 2 wt.% PEG6k and 3 wt.% PEG6k solutions and blends of equal weight concentrations of PEG6k and dextran500k at 2 and 3 wt.%. (**b**) Gain of interfacial energy per volume when surface tension is reduced by Δ*γ* = 0.1 (solid line), 1 (dashed line), and 5 mN/m (dotted line), respectively. (**c**) Schematic illustrations of a polymer blend of short PEG and polydisperse dextran inside a droplet. The upper panel shows the microscopic heterogeneity in the 1-phase region, and the bottom panel shows the membrane wetting–induced 2-phase region. Panels (**a**) and (**b**) are reprinted with permission from Watanabe et al. (2022). Copyright 2022 American Chemical Society.

Another idea is selective wetting for a polymer contained in the blend, where the more strongly wetting polymer spontaneously localizes to the droplet’s surface. Polymers with shorter chains tend to exhibit a higher wettability to the droplet surface due to the fact that shorter chains suffer less entropy loss upon wetting (Mensink, de Beer et al. 2021). To examine this trend, we measured the interfacial tension by using solutions of dextran500k and shorter dextran with a relative molecular weight of 10,000 (dextran10k). As shown Fig. 6) (i), the interfacial tension of PC droplets containing longer dextran500k is greater than that of shorter dextran10k. Additionally, the values of dextran10k, PEG6k, and their combination of dextran500k and dextran10k are comparable to each other (Fig. 5), (ii)). These findings are consistent with the notion that shorter polymers exhibit a higher membrane wettability (Mensink, de Beer et al. 2021). These findings are also consistent with PEG6k’s localization on the droplet surface in the early phase separation stage (Watanabe et al. 2022). The adsorption of PEG to the membrane is congruent with the phenomenon whereby liposomes encapsulating PEG/dextran solutions induce tubular deformation (Crowe and Keating 2018; Liu et al. 2019).

Localization of PEG6k from the homogeneous 1-phase of PEG and dextran blends decreases the interfacial energy at the droplet surface. The degree of decrease in the interfacial energy per unit volume was significantly lower for *R* ≤ 30 μm (Fig. 5). These results suggest that the *R*-dependent phase separation for droplets with *R* ≤ 30 μm is mainly due to the higher membrane wettability of PEG6k. In addition, dextran500k had a greater variance in molecular weight distribution with Mw/Mn ~ 3.1 (Mw and Mn are weight- and number-average molecular weight) than PEG6k Mw/Mn ~ 1.1. It has been reported that near the critical point, dextran of larger molecular weight localizes to the dextran-rich phase (Liu et al. 2019). Such phase separation further promotes the wetting of the membrane by localization of short-chain polymers to the membrane, which may in turn further facilitate phase separation of the system. In summary, *R*-dependent phase separation is likely caused by the high membrane wettability of PEG6k at the membrane surface and heterogeneous nucleation (Fig. 5).

## Concluding remarks and perspective

In this Review I, both introduced and explained the general concept of CSE and described its effects on the phase separation of polymer blends. Although nano-sized spaces (smaller than the critical nucleus size *r*_*c*_) suppress phase separation occurring by a nucleation-growth mechanism, the cell-size space (larger than *r*_*c*_ but smaller than bulk) accelerates and induces such phase separation. The dominant factors during this phase separation are direct and indirect interactions at the droplet surface. The droplet size–dependent phase separation can be explained by the selective wetting of the polymer blends on the cell-size scale. However, the formulation of CSE and its elucidation on a molecular scale remain largely unknown. Further clarification and formulation of CSE are necessary to understand the molecular behaviors inside living cells. Generation of such knowledge may provide an answer as to why cells have a characteristic size on a micrometer-size scale. Furthermore, such knowledge will undoubtedly assist in the generation of artificial cells useful for both cosmetic and biomedical applications.

## The Michèle Auger Award for Young Scientists’ Independent Research

In late 2018, long time Editorial Board Member of *Biophysical Reviews* journal, Professor Michèle Auger, sadly succumbed to illness. As a mark of our respect for Michèle, the *Biophysical Reviews*’ Editorial Board, together with the kind support of Springer-Nature Corporation, created a perpetual memorial award in honor of her life and service. The, “Michèle Auger Award for Young Scientists’ Independent Research,” is granted each year to a single candidate performing biophysical research, who at the time of application is under 40 years of age. The award consists of a plaque and a free personal subscription to the journal along with an invitation to submit a single author review article to *Biophysical Reviews*. This winning review essay for 2022 concludes with two short biographical sketches informing on both this year’s award recipient, Associate Prof. Miho Yanagisawa, and Prof. Michèle Auger, to who’s memory the award is dedicated.

## Biography: Miho Yanagisawa

Prof. Miho Yanagisawa is an experimental physicist in soft matter physics and biophysics who leads a group at the Komaba Institute of Science and the Department of Basic Science at the University of Tokyo. Her group studies the physics of cell-sized soft materials such as polymer droplets, gels, and membrane vesicles and the biophysics of using them as artificial cells.

Miho started her research career in the physics of lipid membranes under the supervision of Prof. Masayuki Imai at Ochanomizu University in Japan. During the Ph.D. course, she elucidated phase separation dynamics and meta-stable patterns in giant liposomes upon lateral phase separation of lipids (Yanagisawa et al. 2007) (Yanagisawa et al. 2008) (Yanagisawa et al. 2010) (Yanagisawa et al. 2012). She explained these phenomena by deriving the dynamic coupling between the phase separation and membrane deformation from experimental observations and theoretical analysis (Taniguchi, Yanagisawa et al. 2011). These results contribute to the physics of phase separation on soft membranes and the biophysics of lipid rafts in biomembranes. Based on these achievements, Miho received the 6th Young Scientist Award from the Physical Society of Japan in 2012.

After receiving her Ph.D. in physics from Ochanomizu University in 2009, Miho conducted post-doctoral research receiving JSPS Research Fellow PD from 2009 to 2011 in the laboratory of Prof. Kenichi Yoshikawa at Kyoto University in Japan. There she focused on molecular behaviors inside cells where various molecules exist at a high concentration and interact with the surrounding membranes such as cellular membranes and membranous organelles. She developed cell-sized polymer droplets coated in a lipid membrane to mimic cytoplasmic circumstances (Fujiwara and Yanagisawa 2014), and she discovered that the phase separation and gene expression are promoted in tiny droplets (Kato et al. 2012).

From 2011 to 2014, Miho was an Assistant Professor in the Department of Physics, Faculty of Science, Kyushu University. She worked in the laboratory of Professor Masayuki Tokita, who specializes in polymer gels. She experimentally found that the cell-size confinement alters coupled dynamics between phase separation and gelation in polymer blends (Yanagisawa et al. 2014a, b, c). However, it was unclear why this behavior changed in a cell-sized space.

Miho approached this longstanding question when she began her independent research as an associate professor at the Tokyo University of Agriculture and Technology from 2014 to 2018 and at the University of Tokyo from 2014. Miho revealed that the direct/indirect membrane effect alters various behaviors (phase separation, gelation (Sakai et al. 2018) (Koyanagi et al. 2019) (Yanagisawa et al. 2018), and other phase transitions, and the accompanying nanostructural transitions of biopolymers and molecular diffusion (Watanabe and Yanagisawa 2018) (Watanabe et al. 2020)) by developing a method to measure molecular behavior in cell-sized space. These results have attracted attention because they provide a possible answer to why cells have a characteristic space size and crowding condition, which can regulate molecular behaviors. Furthermore, she developed methods useful for biophysical research. For example, she established methods for directional reconstitution of membrane proteins into liposomes (Yanagisawa et al. 2011) and for measuring the local mechanics of cells and cell tissues (Sakai et al. 2018) (Sakai et al. 2020). Furthermore, she improved the mechanical level of the liposomes to be able to use as a drug delivery capsule by constructing an artificial DNA cytoskeleton underneath the membrane (Kurokawa et al. 2017). Recently, she has also reported phase transitions in multicellular models consisting of multiple artificial cells (Shinohara et al. 2021) (Fujiwara, Shoji et al. 2020).

Based on these achievements, she received the 1st Fumiko Yonezawa Memorial Prize from the Physical Society of Japan and the Young Scientists’ award from The Minister of Education, Culture, Sports, Science and Technology in 2020. At the time of writing, Miho is very proud to be the recipient of the Michèle Auger Award for Young Scientists’ Independent Research 2022 based on the results of the above independent research.

In the Global Gender Gap Report, Japan is ranked 120th in 2021 and 116th in 2022. She regrets this situation in Japan and has contributed to improving gender quality at affiliated universities, biophysical societies, and physics societies. Furthermore, she dedicates herself to providing a more comfortable and stimulating environment for women and gender minorities in science and engineering.

Miho likes ceramics, especially those rooted in daily life, called Mingei in Japanese (folk art). For instance, to create the exquisite colors of the salt glaze, sodium and silicic acid are combined in earthenware. She likes to find the marriage of science and beauty in everything she encounters in her daily life. Such attitude connects to her specialty in soft matter physics and biophysics. Nevertheless, it is not easy to continue both as a researcher and as a mother with childcare responsibilities. Therefore, I am deeply grateful for the understanding and support of my partner Kei Fujiwara, an associate professor of synthetic biology, and my 6-year-old daughter Honoka Fujiwara.
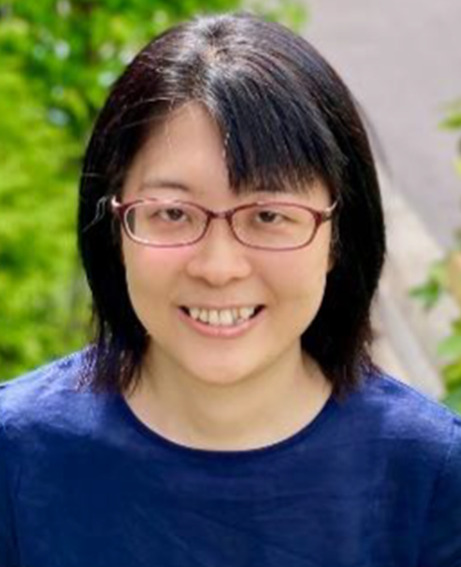


## Biography: Michèle Auger (1963 – 2018)

Born in GrandMère, Quebec and raised in TroisRivières, Michèle Auger enrolled first in biophysics at the Université du Québec à Trois-Rivières, only to later transfer to chemistry. After obtaining her B. Sc. in 1985, Michèle joined the group of Prof. Ian C.P. Smith at the University of Ottawa/National Research Council of Canada to pursue her Ph.D. studies in biophysics. After graduating from the University of Ottawa in 1990, Michèle refined her skills in solid state NMR as a postdoctoral fellow in the group of Prof. Robert G. Griffin at the Massachusetts Institute of Technology. Michèle joined the Department of Chemistry at the University of Laval in 1991 as an Assistant Professor and recipient of an NSERC Women’s Faculty Award. She was promoted to Associate Professor in 1996 and then Professor in 2000 where she remained until 2018. Michèle’s research involved using solid state NMR to study (i) the interaction of proteins, peptides and drugs with phospholipid membranes, and (ii) biopolymers such as spider silk. Michèle served internationally on the Council of the International Union of Pure and Applied Biophysics from 2011 – 2017 and was an Editorial Board Member of Biophysical Reviews journal from 2011-2018. Brilliant, creative, dedicated to the scientific and academic communities, Michèle displayed admirable professional, ethical and leadership qualities. She is remembered as a dedicated, generous, and inspirational scientist who touched the lives of many through her friendship, teaching and kindness.

Professor Michèle Auger a much admired Editorial Board Member of Biophysical Reviews (2011 – 2018)


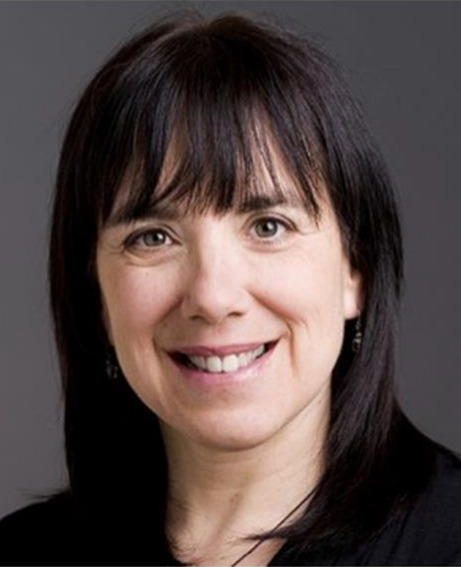


(This short foreword is adapted from a longer memorial published on the IUPAB Newsletter (IUPAB 2019).
